# Tridimensional Personality Questionnaire data on alcoholic violent offenders: specific connections to severe impulsive cluster B personality disorders and violent criminality

**DOI:** 10.1186/1471-244X-7-36

**Published:** 2007-07-30

**Authors:** Roope Tikkanen, Matti Holi, Nina Lindberg, Matti Virkkunen

**Affiliations:** 1 Department of Psychiatry, University of Helsinki, PO Box 590, Helsinki, Finland

## Abstract

**Background:**

The validity of traditional categorical personality disorder diagnoses is currently re-evaluated from a continuous perspective, and the evolving DSM-V classification may describe personality disorders dimensionally. The utility of dimensional personality assessment, however, is unclear in violent offenders with severe personality pathology.

**Methods:**

The temperament structure of 114 alcoholic violent offenders with antisocial personality disorder (ASPD) was compared to 84 offenders without ASPD, and 170 healthy controls. Inclusion occurred during a court-ordered mental examination preceded by homicide, assault, battery, rape or arson. Participants underwent assessment of temperament with the Tridimensional Personality Questionnaire (TPQ) and were diagnosed with DSM-III-R criteria.

**Results:**

The typical temperament profile in violent offender having ASPD comprised high novelty seeking, high harm avoidance, and low reward dependence. A 21% minority scored low in trait harm avoidance. Results, including the polarized harm avoidance dimension, are in accordance with Cloninger's hypothesis of dimensional description of ASPD. The low harm avoidance offenders committed less impulsive violence than high harm avoidance offenders. High harm avoidance was associated with comorbid antisocial personality disorder and borderline personality disorder.

**Conclusion:**

Results indicate that the DSM based ASPD diagnosis in alcoholic violent offenders associates with impulsiveness and high novelty seeking but comprises two different types of ASPD associated with distinct second-order traits that possibly explain differences in type of violent criminality. Low harm avoidance offenders have many traits in common with high scorers on the Hare Psychopathy Checklist-Revised (PCL-R). Results link high harm avoidance with broad personality pathology and argue for the usefulness of self-report questionnaires in clinical praxis.

## Background

The antisocial personality disorder diagnosis (ASPD) is frequent in prison populations [1], and prominent in assessment of violence. The validity of ASPD diagnosis, however, is unclear. The descriptive categorization criteria (present/absent) for ASPD were introduced in DSM-III [2]. Recently, Widiger and co-workers summed up personality disorder research and proposed a research agenda for the DSM-V that advocates replacing the current categorical approach, including ASPD, with a dimensional approach that would treat personality traits as continua [3].

Critics of diagnostic dichotomies state that categorical diagnoses are neither reliable nor valid [4], because severely ill patients have abundantly overlapping diagnoses [5]. Another validity issue is whether categorical diagnoses connect to the causes of personality disorders. The etiology of cluster B disorders, particularly borderline personality disorder (BPD) and ASPD, seems to be a dynamic field of research; phenotypes are no longer predestined expressions of a gene or an environmental factor, but rather a result of complex gene – environment interactions [6,7]. In favour of dimensionality, the biologic etiology of the categorical BPD diagnosis, for instance, is explicitly described in terms of dimensional traits [8].

Cloninger proposed a model for assessing personality in a continuous tridimensional space. He hypothesized that the extreme variants of hereditary temperament traits predispose to clinical personality disorders. He claimed that ASPD would typically feature high novelty seeking, low harm avoidance, and low reward dependence [9].

This paper outlines the temperament profile of alcoholic violent offenders and aims to test (a) Cloninger's hypothesis of dimensional temperament deviation in alcoholic ASPD, and (b) its connection to severe violence.

## Methods

### Participants

Participants comprised 198 male violent offenders recruited between 1990 and 1998 from a court-ordered, two-month in-patient mental examination, at the Department of Forensic Psychiatry of Helsinki University Central Hospital. The study-sample included many offenders who participated in recent studies concerning Finnish alcoholic offenders, mainly with ASPD [10-12]. Violent offenses were generally serious, impulsive, and committed under the influence of alcohol.

Controls were 170 healthy age- and gender-matched volunteers recruited through newspaper advertisements and they underwent the same TPQ and lifetime DSM-III-R evaluation as the offenders. Originally 181 responders were interviewed. Eleven (6%) were excluded due to diagnosed major depression, dysthymia, panic disorder, or borderline personality disorder. The occupations of the controls were longshoremen, firemen, postmen, policemen, vocational students, and a few university students. The offenders had a similar education and work history but were more frequently unemployed.

### Instruments and procedure

Each participant was interviewed with the DSM-III-R [2] Structured Clinical Interview [13] for ASPD and other mental disorders. Interviewers were experienced licensed psychiatrists.

The assessment of temperament traits was performed with Cloninger's Tridimensional Personality Questionnaire (TPQ), a 100-item questionnaire that measures three genetically distinct personality dimensions: novelty seeking (NS), harm avoidance (HA), and reward dependence (RD), each of which consists of four lower-order dimensions [14] that are shown in additional file 1. It is a self-report questionnaire that takes 20–30 minutes to complete. In order to decrease mitigating answers the total question-set contains 17% non-scored questions. The validity of the questionnaire seems to be good according to a large psychometric investigation in the Finnish general population [15]. The present study gives information of the usefulness of TPQ in Finnish alcoholic offenders.

The offenders were primarily divided into two groups, ASPD and non-ASPD, before statistical analysis and pairwise comparison. Secondarily, after discovering marked polarisation of dimension HA, they were divided into low HA (score 11 or smaller) and high HA (12 or higher) groups. The cut-off point was chosen on the basis of the control mean score (10.9 SD 5.4) and alcohol-genetic research associating low/high HA (with the same cut-off point) to different haplotypes of genes [10,11].

The study was approved by the Ethics board of Helsinki University Central Hospital.

### Statistics

The variance analysis of TPQ mean scores was performed with the Kruskal-Wallis test and pairwise comparisons with the Dunn's test. These conservative non-parametric methods were applied to reduce the possibility of type I error, as all dimensions were skewed or kurtotic, or both. The Kolmogorov-Smirnov method served to test the normality of the data. The Pearson Chi-Square and Fisher's exact tests were applied in frequency distribution comparisons of diagnoses, marital status, occupation, and types of crime. ANOVA was applied to compare the variance of age. Analyses were performed with SPSS 14.0.

## Results

### Potential confounding factors

Mean age was 30.7 years in the ASPD group (SD 8.6, 18–52), 35.7 in the non-ASPD group (SD 10.4, 18–67), and 30.7 in the control group (SD 9.9, 18–59); F(1214, 178) = 6.387, p = .002.

The frequency distribution of offenses was as follows: homicide (n = 99; 50%), attempted homicide (39; 20%), assault-battery-robbery as a group (18; 9%), arson (34; 17%), and rape (8; 4%). There were no significant differences in offenses between the ASPD and non-ASPD groups, except for arson that was more prevalent in the non-ASPD group; χ^2 ^(1) = 16.388, p < .0004. Criminal record excerpts revealed that the offenders' criminal careers featured, at inclusion, a mean of 11 lifetime convictions (including non-violent crimes).

The SCID I and SCID II diagnoses and a comparison of them between ASPD and non-ASPD participants are presented in Table 1. The ASPD group (114 out of 198; 58%) seemed to comprise more BPD than the non-ASPD group but the difference was non-significant (p=.06). The sample comprised 50 (25%) participants with an ASPD-BPD comorbidity.

**Table 1:** Mental disorders of violent offenders.

	**All (n = 198)**	**ASPD + (n = 114)**	**ASPD - (n = 84)**	**t**	**p**
**SCID-I diagnoses:**					
Alcohol dependence	171 (86%)	106 (93%)	67 (80%)	8.952	.003
Anxiety disorder	10 (5%)	4 (4%)	6 (7%)	1.661*f*	.254
Mood disorder	39 (20%)	24 (21%)	15 (18%)	.049	.825
**SCID-II diagnoses:**					
Two or more PD	86 (44%)	74 (65%)	12 (14%)	63.726	.000
Borderline PD	77 (39%)	50 (44%)	27 (32%)	3.472	.062
Narcissistic PD	10 (5%)	7 (6%)	3 (3%)	1.348*f*	.355
Paranoid PD	28 (14%)	17 (15%)	11 (13%)	.301	.583

Pairwise comparison between offenders with and without antisocial personality disorder (ASPD+/-). *f *= Fisher's Exact Test was applied where a cross table cell contained less than 5 observations; Pearson's Chi-Square test was used for other comparisons. All comparisons were carried out with 1 degree of freedom. PD, personality disorder.

There was no difference in occupation between ASPD vs. non-ASPD (χ^2 ^(1) = .426, p = .422) and high- vs. low HA (χ^2 ^(1) = .344, p = .498) offenders. Participants were equally frequent divorced or unmarried in the ASPD vs. non-ASPD (χ^2 ^(1) = .663, p = .468) and high- vs. low HA (χ^2 ^(1) = .001, p = 1.0) groups.

### TPQ scores and pairwise comparisons

The TPQ higher-order means of the entire offender sample were as follows: NS 18.7 (SD 4.8), HA 17.9 (SD 6.8), and RD 15.5 (SD 4.9). TPQ mean scores and contrasts of the ASPD, non-ASPD, and control groups are displayed in Additional file 1.

The significant differences (p-values) in a pairwise comparison between ASPD vs. non-ASPD offenders were as follows: NS .000; NS2 .006; NS4 .000.

The significant differences between ASPD offenders and controls were: NS .000; NS2 .000; NS3 .004; NS4 .000; HA .000; HA1 .000; HA2 .000; HA3 .000; HA4 .000; RD .005; RD3 .000; RD4 .001.

The significant differences in pairwise comparison between non-ASPD offenders and controls were as follows: NS1 .005; NS3 .008; HA .000; HA1 .000; HA2 .000; HA3 .000; HA4 .000; RD1 .036; RD3 .002.

### Features of the harm avoidance extreme-groups

Trait HA was particularly non-normally distributed. A scatter-plot displayed a two-peaked curve with a kurtosis of -.766 (SE .346). In other words, on the HA scale (0–34), there appeared a notable over-representation of scores between 5 and 10, and between 25 and 30, whereas an under-representation occurred between 10 and 15.

**Figure 1:**
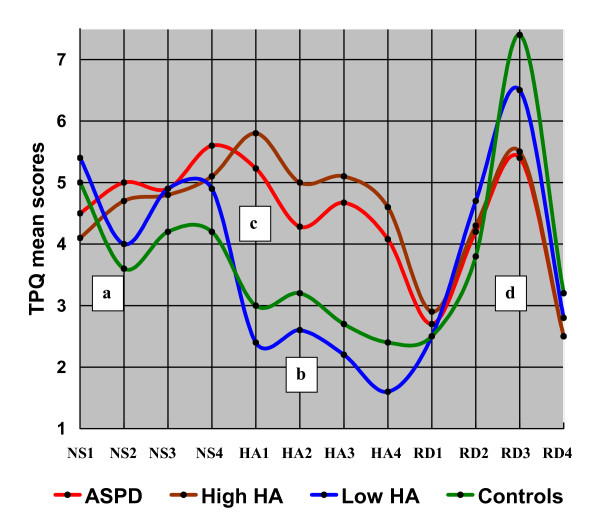
**Extreme Harm Avoidance (HA) temperament and antisocial personality disorder (ASPD) profiles in violent offenders and controls**. Violent offenders were divided into those with high HA and those with low HA on the basis that 21% (42/198) of offenders scored lower or equal to control HA mean. The temperament profile of offenders with low trait harm avoidance followed the control profile and deviated substantially (SD 2.29) from the high HA offender mean. The mean profile of the high HA offenders resembled that of the offenders with antisocial personality disorder. A) Displays that (1) low HA offenders showed no difference compared to controls in trait impulsiveness (NS2); p = .85 (absolute mean rank difference = 19.8), whereas high HA offenders scored higher than controls on trait impulsiveness; p = .0004 (52.7), (2) low HA offenders featured more curiosity and excitement (NS1) than high HA offenders; p = .0001 (78.7), and (3) the total novelty seeking score showed no difference between high and low HA groups; p = 1.0 (14.1). B) Displays that low HA offenders' HA mean score was even lower than the mean score of the lower half of controls; 7.7 SD 3.1 vs. 9.2 SD 2.5; p = .002 (23.0). C) Trait HA1 showed the largest absolute score difference between low and high HA offenders (3.4) indicating that low HA offenders were more optimistic and careless than high HA offenders who were prone to anticipatory worry and anxiety. D) Low HA offenders were dependent on environmental reward (RD3) similarly to controls; p = .12 (42.3), whereas high HA offenders featured greater detachment and individualism than controls; p = .0004 (75.8). Kruskal-Wallis test (df = 2) was applied for comparison of means.

Figure 1 shows the TPQ profiles of high and low HA offenders, ASPD offenders, and controls.

Murder convictions were more frequent in the low HA offenders; χ^2 ^(1) = 4.025, p = .04. Assault- and battery convictions were more frequent in high HA offenders; χ^2 ^(1) = 4.07, p = .03. There were no differences between groups in other violent offenses.

The frequency distribution of the ASPD diagnosis showed no difference (χ^2 ^(1) = .367, p = .55) between high- (60%) and low (55%) harm avoidance offenders. Nineteen low HA offenders lacked an ASPD diagnoses. There were more ASPD and BPD co-morbidities in the high HA group, χ^2 ^(1) = 6.039, p = .009. No difference in alcohol dependence occurred between the HA extremes; χ^2 ^(1) = .98, p = .23.

The mean age of the low HA group was 31.2 years (SD 9.8, 18–53), and 33.6 (SD 9.6, 18–67) in the high HA group; F(206,6) = 2.205, p = .14.

## Discussion

In line with our hypothesis the TPQ profile of violent ASPD mostly suits Cloninger's definition of ASPD. We also hypothesized that ASPD distinguishes types of violent crimes. Results suggest that arson is more common in the non-ASPD group than among offenders who had ASPD but no other differences occurred.

The typical violent offender temperament profile comprised high NS, high HA, and low RD. This profile suits Cloninger's proposal of explosive personality. Further, an explosive personality linked to alcoholism and antisocial behavior – Cloninger claims – corresponds with the impulsive-aggressive antisocial personality disorder described in DSM-III, distinct from his definition of antisocial personality disorder ("primary psychopath") with high NS (including second order traits of impulsive-aggressive behavior), low HA, and low RD [9]. Our results support this kind of distinction among alcoholic persons with ASPD because we observed, embedded in the general high harm avoidance trend, a notable offender minority with exceptionally low harm avoidance scores. Results suggest that low HA connects to ASPD on its own, whereas high HA corresponds with the comorbidity of ASPD and borderline personality disorder. This suits Cloninger's hypothesis as well. Though, in contrast to Cloninger, our "primary psychopaths" who featured low HA, connected to second-order traits of low impulsiveness (NS2) and high degree of social attachment (RD3) similar to the traits of our controls. We note that 45% of the low HA offenders lacked an ASPD diagnosis.

According to Cloninger, low HA manifests a temperament of uninhibited optimism, confidence, gregariousness, and vigor [9] that presumably results in character features of excellent consecutive behavioral ability. Taking this further, and combining it with our finding of low impulsiveness in low HA offenders and elevated impulsiveness in high HA offenders we argue that low HA offenders are prone to commit premeditated violent crimes (murders in our sample) whereas impulsive violence (assaults and batteries) connects to the high HA offenders.

The temperament features of the low harm avoidance subpopulation springs a hypothesis that low HA may correlate with high factor-1 scores in Hare's Psychopathy Checklist-Revised [16], as these seem to have many nuances in common: (1) gregariousness/vigor (TPQ) versus glibness/superficial charm (PCL-R), (2) uninhibited optimism versus a grandiose sense of self-worth/confidence, and (3) a carefree attitude versus shallow affect. Moreover, we observed a high resemblance in the total temperament profiles of controls and low HA offenders implying that low HA offenders may respond to stimulus in a relatively normal manner. Studies combining TPQ and PCL-R are necessary to test this hypothesis.

Our observation of high harm avoidance in these alcoholic offenders with severe personality is in line with research that suggests an association of a broad spectrum of specific personality disorders with high HA temperament [17,18], and recently with comorbid alcoholism and ASPD, as well [10,11]. Preliminary genetic polymorphism discoveries in alcohol research show that GABRA2 haplotype- [11] and galanin haplotype [10] frequencies differ in low and high HA alcoholics. In this study, however, the HA extremes showed no difference in clinical alcohol dependence but rather a difference in severe impulsive cluster B psychopathology. All offenders in this study suffered from an alcohol usage disorder, in contrast to alcohol studies in which alcoholics are compared to non-alcoholics. Severe impulsive cluster B personality disorders and alcoholism may have an additive association to deviation in trait HA.

In line with Cloninger's hypothesis, we found that trait novelty seeking was higher in ASPD than in non-ASPD offenders. Similar results have emerged for substance users [18], and psychiatric patients with cluster B disorders [17]. Moreover, ASPD offenders showed particularly high impulsiveness (NS2) and disorderliness (NS4), whereas non-ASPD offenders corresponded to the control figures in these dimensions. These associations are comprehensible since the TPQ surveys features that are similar to the features surveyed in the ASPD and BPD sections of the structured interview for DSM-III-R [2]. All 8 NS2 items survey impulsiveness with questions such as "I often act on hunches, momentary whims, or by intuition without making a detailed analysis of facts" [9]. The 10 NS4 items survey issues such as breaking rules, lying, and losing one's temper [14]. Impulsiveness (NS2) and disorderliness (NS4) are likely to be linked to biologic correlates of ASPD. Brain imaging studies strongly suggest that among habitually violent offenders with ASPD, impulsive aggressive behavior is associated with decreased glucose uptake in the frontal cortex (reviewed by Bufkin & Luttrell, 2005 [19]), and that a low brain serotonin turnover, indicated by low cerebrospinal fluid 5-hydroxyindoleacetic acid (CSF 5-HIAA) levels, is also connected with impulsive aggression [19,20]. Our preliminary observation of NS1 suggests that high HA offenders are more indifferent to stimulus than the low HA offenders.

Examining the third dimension, ASPD offenders featured low reward dependence and considerable detachment (RD3) compared to controls in agreement with Cloninger's hypothesis, but no difference to offenders lacking ASPD emerged. Trait harm avoidance, however, distinguished offenders with higher reward dependence (low HA offenders) from their counterparts.

To the best of our knowledge, this is the first systematic study of temperament traits in alcoholic violent offenders in relation to categorical ASPD diagnosis and the type of violent offense. The strengths of this study comprise its large sample size, accurate diagnoses, and simple study design. The limitations, on the other hand, were that participants were a selected group of males who featured substantial psychopathology. Consequently, results may not be generalized to other patient groups such as the ASPD patients with infrequent or unregistered minor property crimes. Secondly, we chose the conservative Kruskal-Wallis and Dunn's tests for variance and pairwise comparisons due to the non-normally distributed data and unequal group sizes, which left potential confounding factors uncontrolled for (for instance age). However, our population is markedly homogeneous compared to community based settings were demographic data is more relevant. According to Cloninger's original proposal, the TPQ dimensions are hereditary, and stable basic stimulus-response characteristics, which, being true, also decreases the risk for bias due to demographic differences. Another source of bias is the temperament figures of our control group since curious and novelty-seeking individuals may be over-represented in control groups attracted by newspaper advertisements [21], but still, the TPQ scores were in line with the results of a large psychometric TPQ testing project in the Finnish population [15]. Inter-rater agreement analysis was not performed, which is a limitation even though we consider the SCID administration in the unit as well standardized due to frequent administration by experienced psychiatrists and the diagnoses were double-checked by other psychiatrists. Moreover, ASPD offenders were more frequently alcohol dependent than non-ASPD offenders. On the other hand the non-ASPD offenders who lacked an alcohol dependence diagnosis suffered from severe alcohol abuse. This difference in alcohol-disorders could be a source of bias even though the overall contrast to the controls was robust, as any substance usage disorder was an exclusion criterion for controls. Finally, every fifth offender chose not to complete a TPQ (resulting in the sample of 198 offender analysed in this paper) and thus, returned questionnaires could be biased due to the desire for judiciary benefit. The non-returnees had no obvious differences compared with other offenders in this homogeneous sample.

## Conclusion

We expect our findings to contribute to the discussion concerning dimensional versus categorical diagnostics of personality disorders. Results suggest some advantages of dimensional assessment of temperament over the traditional ASPD versus non-ASPD dichotomy in alcoholic violent offenders; results suggest that the harm avoidance extremes distinguish two types of antisocial personality disorder and offenders who are prone to commit different types of violence. The preliminary findings presented in this paper, however, requires further study.

## Competing interests

The author(s) declare that they have no competing interests.

## Authors' contributions

RT organized data, analyzed data, and served as first author.

MH contributed with ideas and supervised statistical analysis.

NL participated in the writing process.

MV provided the material, contributed with ideas and context, and supervised the whole project.

All authors read and approved the final manuscript.

## Pre-publication history

The pre-publication history for this paper can be accessed here:



## Supplementary Material

Additional file 1 **TPQ mean scores (SD) among 198 alcoholic violent offenders**. Pairwise comparison and contrasts between antisocial personality disorder (ASPD+; n = 114), violent offenders without it (ASPD-; 84), and healthy controls (c; 170). The Kruskal-Wallis test was used for analysis of variance and the Bonferroni corrected Dunn's test for multiple comparisons. Significance level p < .01 was applied for contrasts; degree of freedom at 2 for all dimensions. Click here for file
