# Supplementary material for: Tridimensional Personality Questionnaire data on alcoholic violent offenders: specific connections to severe impulsive cluster B personality disorders and violent criminality
Source: BMC Psychiatry. 2007 Jul 30;7:36. doi: 10.1186/1471-244X-7-36 (PMC1976096; doi:10.1186/1471-244X-7-36)
Supplement: Additional file 1 — TPQ mean scores (SD) among 198 alcoholic violent offenders. Pairwise comparison and contrasts between antisocial personality disorder (ASPD+; n = 114), violent offenders without it (ASPD-; 84), and healthy controls (c; 170). The Kruskal-Wallis test was used for analysis of variance and the Bonferroni corrected Dunn's test for multiple comparisons. Significance level p < .01 was applied for contrasts; degree of freedom at 2 for all dimensions. [file 1471-244X-7-36-S1.doc]

| **Temperament traits; high – low** |  | **ASPD+** | **ASPD–** | **Control** | **χ2** | **p** | **Contrasts** |
| --- | --- | --- | --- | --- | --- | --- | --- |
| **Novelty seeking** | **NS** | 20.0 (4.4) | 17.2 (5.1) | 17.0 (4.5) | 28.5 | .000 | ASPD+ > ASPD- and C |
| **Exploratory excitability – stoic rigidity** | **NS1** | 4.5 (1.7) | 4.1 (1.8) | 5.0 (1.8) | 12.7 | .002 | **ASPD- < C** |
| Impulsiveness – reflection | **NS2** | 5.0 (2.1) | 3.9 (2.2) | 3.6 (2.0) | 26.9 | .000 | ASPD+ > ASPD- and C |
| **Extravagance – reserve** | **NS3** | 4.9 (1.5) | 4.9 (1.7) | 4.3 (1.4) | 19.5 | .000 | **ASPD+/- > C** |
| **Disorderliness – regimentation** | **NS4** | 5.6 (1.9) | 4.4 (2.1) | 4.2 (1.6) | 35.8 | .000 | ASPD+ > ASPD- and C |
| **Harm avoidance** | **HA** | 18.2 (6.9) | 17.7 (6.8) | 10.9 (5.4) | 90.8 | .000 | **ASPD+/- > C** |
| Anticipatory worry – uninhibited optimism | **HA1** | 5.2 (2.4) | 4.8 (2.3) | 3.0 (2.0) | 67.7 | .000 | **ASPD+/- > C** |
| **Fear of uncertainty – confidence** | **HA2** | 4.3 (2.0) | 4.7 (1.7) | 3.3 (1.4) | 45.3 | .000 | **ASPD+/- > C** |
| **Shyness with strangers – gregariousness** | **HA3** | 4.7 (2.0) | 4.2 (2.2) | 2.3 (1.8) | 92.7 | .000 | **ASPD+/- > C** |
| Fatigability and asthenia – vigor | **HA4** | 4.1 (2.6) | 3.9 (2.7) | 2.4 (2.0) | 35.9 | .000 | **ASPD+/- > C** |
| Reward dependence | **RD** | 14.7 (4.8) | 16.5 (4.9) | 16.8 (4.0) | 14.4 | .001 | **ASPD+ > C** |
| **Sentimentality – insensitiveness** | **RD1** | 2.7 (1.5) | 3.0 (1.3) | 2.5 (1.2) | 8.3 | .016 | **ASPD- > C** |
| **Persistence – irresoluteness** | RD2 | 4.2 (1.9) | 4.7 (1.9) | 3.8 (1.8) | 9.5 | .009 | **ASPD- > C** |
| **Attachment – detachment** | **RD3** | 5.4 (2.6) | 6.2 (2.7) | 7.4 (2.5) | 42.5 | .000 | **ASPD+/- < C** |
| **Dependence – independence** | **RD4** | 2.5 (1.4) | 2.8 (1.4) | 3.1 (1.3) | 19.0 | .000 | **ASPD+ < C** |
